# Navigating challenges: optimising methods for primary cell culture isolation

**DOI:** 10.1186/s12935-023-03190-4

**Published:** 2024-01-11

**Authors:** Oliwia Piwocka, Marika Musielak, Karolina Ampuła, Igor Piotrowski, Beata Adamczyk, Magdalena Fundowicz, Wiktoria Maria Suchorska, Julian Malicki

**Affiliations:** 1 Department of Electroradiology, Poznan University of Medical Sciences, Poznan, 61-701 Poland; 2 Doctoral School, Poznan University of Medical Sciences, Poznan, 61-701 Poland; 3 Radiobiology Laboratory, Department of Medical Physics, Greater Poland Cancer Centre, Poznan, 61- 866 Poland; 4 Faculty of Biology, Adam Mickiewicz University, Poznan, 61-614 Poland; 5 Breast Surgical Oncology Department, Greater Poland Cancer Centre, Poznan, 61-866 Poland; 6 Radiotherapy Ward I, Greater Poland Cancer Centre, Poznan, 61-866 Poland; 7 Medical Physics Department, Greater Poland Cancer Centre, Poznan, 61-866 Poland

**Keywords:** Primary culture, Breast cancer, Isolation, Enzymatic digestion, Cancer cells, Cancer-associated fibroblasts, Primary cell line

## Abstract

Primary cell lines are invaluable for exploring cancer biology and investigating novel treatments. Despite their numerous advantages, primary cultures are laborious to obtain and maintain in culture. Hence, established cell lines are still more common. This study aimed to evaluate a range of techniques for isolating primary breast cancer cultures, employing distinct enzymatic compositions, incubation durations, and mechanical approaches, including filtration. Out of several protocols, we opted for a highly effective method (Method 5) that gave rise to a primary cell culture (BC160). This method combines mechanical disaggregation and enzymatic digestion with hyaluronidase and collagenase. Moreover, the paper addresses common issues in isolating primary cultures, shedding light on the struggle against fibroblasts overgrowing cancer cell populations. To make primary cell lines a preferred model, it is essential to elaborate and categorise isolation methods, develop approaches to separate heterogeneous cultures and investigate factors influencing the establishment of primary cell lines.

**Supplementary Information:**

The online version contains supplementary material available at 10.1186/s12935-023-03190-4.

## Background

Breast cancer (BC) is the most common neoplasm in women, responsible for 2,261,419 deaths in 2020, which makes it the highest mortality due to cancer in both developed and developing countries [1, 2]. Researchers predict decreasing mortality of breast cancer patients [3, 4] due to improvements in treatment methods, advancements in breast screening, and higher awareness among women [5]. During the last decades, the research on breast cancer was seminal for the development of innovative treatments for breast cancer. The elucidation of interpersonal genetic variations and functional characteristics with the help of primary cancer cell lines has been an important pre-requisite for this progress [6].

A primary cell line refers to a culture of cells directly derived from tissues or organs of an organism. These cells are typically used in laboratory research to study various aspects of cell biology, physiology, and disease. Unlike immortalized cell lines, which can divide indefinitely, primary cell lines have a limited lifespan in culture, reflecting the normal biological constraints of the cells [6]. Primary cancer cells can be obtained from surgically resected tissue samples, core biopsies, aspirates, pleural effusions, and autopsy materials. Cell populations can differ depending on the isolation method and the tissue composition of the collected material. Moreover, cells of the tumour microenvironment (TME) such as normal fibroblasts (NFs), cancer-associated fibroblasts (CAFs), endothelial cells, macrophages and lymphocytes, and endothelial cells are a crucial part of the experimental system [7].

Tumour resections and tumour biopsies are the favoured source for primary cultures [6, 8–13]. Primary cultures derived from tumour tissue preserve characteristics of the TME and the cells’ stem-like phenotype and display a specific cross-talk between healthy and malignant cells [14]. This intercellular cross-talk is critical during carcinogenesis, progression, and invasion and plays a vital role in response to therapy [15]. These features assure the validity of primary cultures as a model for preclinical studies or designing personalised therapy since patient-derived primary cells reflect tumour nature more accurately than immortalised cell lines [14]. Another advantage of primary cultures is a large transcriptomic and proteomic variety, which is important for personalized medicine research [16]. Moreover, primary cells preserve cellular markers and tissue functionality, while established cell lines often lose those properties [14]. Limitations of primary cell cultures are difficult isolation, short lifespan, and a finite ability to replicate [17]. Although established cell lines enable researchers to work on the same material worldwide, which guarantees replicability, and that feature has contributed to the formulation of many currently used therapies, primary cells are the better model according to individual requirements [14].

This article aimed to show the efficacy of different enzymatic and mechanical isolation methods for primary cancer cells applicable to breast cancer tumour biopsies. This paper systematises the current knowledge and protocols of isolation of primary BC cells and thus may serve as a compendium for this area of research. Moreover, the article addresses common issues in isolating primary cultures, shedding light on the struggle against fibroblasts overgrowing cancer cell populations.

## Methods

### Collection of breast cancer biopsies

Thirty patients with a histological diagnosis of invasive BC (diameter ≤ 15 mm) qualified for surgical treatment were recruited for the study. The core needle biopsies and breast skin samples were collected with the patient’s consent by the surgeon and were placed in a tube with a sterile medium to be delivered to the Radiobiology Department. Ethical approval for the study (number 283/21) was obtained from the Bioethics Committee of Poznan University of Medical Sciences. All experiments were performed following relevant guidelines. Written consent was obtained from all the participants, who were informed about the study’s purpose, risks, and benefits.

### Pathological review

Pathologists examined the specimens of BC tissue to determine their morphological and immunocytochemical characteristics. A haematoxylin-eosin (H&E) staining was performed to determine the shape and structure of cells, and antibody-coupled staining was used for the analysis of cytokeratin 7 (CK7), cytokeratin 20 (CK20), mammaglobin, Gross Cystic Disease Fluid Protein-15 (GCDFP15), oestrogen receptor (ER), progesterone receptor (PR), and human epidermal growth factor receptor 2 (HER2).

### Isolation of primary breast cancer cells

Five different methods for isolating primary BC cells were chosen. To optimise the isolation process, 1 sample of BC biopsy (size approx. 10 mm) from 3 patients for each isolation method (15 samples) was used. The approaches involved enzymatic digestion, mechanical disaggregation, and centrifugation combined or applied separately in various variants or with different types of enzymes. After optimising and choosing a protocol for isolation, we isolated the primary cell lines from 15 BC biopsy samples using this method.

#### Method 1

Tissue samples were minced with a scalpel before enzymatic digestion. For enzymatic disaggregation, we initially incubated with 1 mg/mL collagenase IV (Thermo Fisher Scientific, France) for 1 h, 2.5 h, and 24 h. To shorten incubation time, we introduced trypsin. Subsequently, digestion with 1 mg/mL collagenase IV was combined with 0.25% trypsin-EDTA (Sigma Aldrich, MO, USA): 1 h collagenase with 5 min trypsin, 2 h collagenase with 2 min trypsin.

#### Method 2

**Fig. 1:**
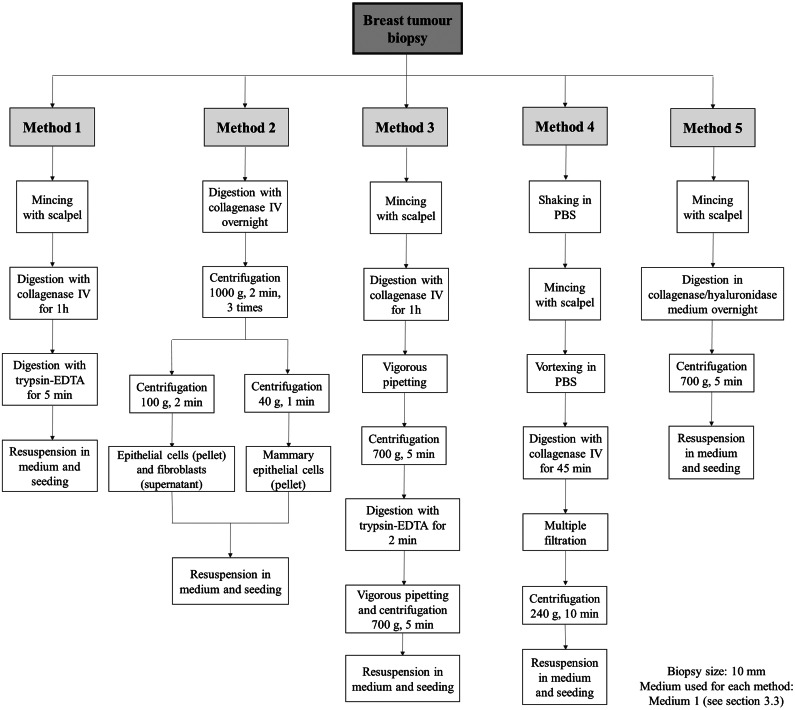
A comparison of the different protocols used in the study. Methods 1–5 differ in digestion times, the type and concentration of enzymes, and the centrifugation methods

Based on the method described by Faridi et al., we established Method 2, which is based on a combination of enzymatic digestion and differential centrifugation [18]. Firstly, the samples were digested with 1 mg/mL collagenase IV at 37 °C overnight. Enzymatically treated specimens were washed with medium, followed by PBS (Biowest, France), and centrifuged three times at 1000 g for 2 min. Cells were resuspended between each centrifugation cycle. Next, the cell suspension underwent centrifugation at 100 g for 2 min to obtain the pellet rich in epithelial cells, which was washed with medium and seeded. The remaining supernatant was transferred to wells to obtain a fibroblast population. The original protocol also includes centrifugation at 40 g for 1 min to obtain organoid fraction, however, organoids were not in the scope of our interest (Fig. 1).

#### Method 3

Method 3 represents combines enzymatic digestion with mechanical methods [19]. The tissue sample was cut with a scalpel and incubated in 1 mg/mL collagenase IV solution for 1 h at 37 °C. After digestion, the cell suspension was vigorously pipetted until there were no tissue clumps and the suspension was as homogenous as possible. Then, the tissue slurry was suspended in PBS and centrifuged at 700 g for 5 min. The supernatant was removed, and the pellet was resuspended in 1 mL of 0.25% trypsin-EDTA solution for 2 min at 37 °C. After incubation, the cell suspension was pipetted and centrifuged again. The pellet was washed twice with PBS and resuspended in the medium. The suspension was then seeded onto wells and cultured.

#### Method 4

The tissue obtained during the BC biopsy was placed on the Petri dish, washed twice with PBS, and shaken firmly [20]. Then, tissue was dissected with the scalpel to obtain a homogeneous slurry, which was later immersed in PBS and vortexed. When pieces of tissue settled down, the supernatant was aspirated, and the remaining tissue was transferred to collagenase IV for 45 min at 37 °C. After digestion, the cell suspension was filtered through filters with membrane pore size 75 μm (BD Becton Dickinson, New Jersey, USA). Filtering was followed by centrifugation at 240 g for 10 min. The supernatant was discarded, and the pellet was seeded onto a 12-well plate (Corning Inc., NY, USA).

#### Method 5

The tissue obtained during the BC biopsy was cut into small pieces of 1 mm^3^ in volume and then placed in 1 mL of digestion medium for overnight incubation [21]. The digestion medium consisted of DMEM, antibiotic agents penicillin/streptomycin at a final concentration of 1%, 0.14 mg/mL of hyaluronidase (Thermo Fisher Scientific, France), and 1.6 mg/mL collagenase IV. After incubation, the suspension was transferred to the tube containing 2 mL PBS. The tissue slurry was centrifuged at room temperature at 700 g for 5 min. Afterward, the supernatant was removed, the pellet was resuspended in the fresh culture medium, and seeded on 3 wells of a 12-well plate.

#### Isolation of normal primary fibroblasts

Normal fibroblasts were derived from the skin of the same breast from which the biopsy was taken. The tissue sample was minced with a scalpel and digested in 2 mg/mL collagenase IV, 2 mg/mL hyaluronidase, 2 mg/mL Bovine Serum Albumin (BSA) (Sigma Aldrich, MO, USA), 1% penicillin/streptomycin (P/S) in DMEM medium overnight. The obtained tissue slurry was washed with PBS and seeded onto 10 cm culture plates. For the purpose of this study, we used the primary normal fibroblast cell line NF160, which was isolated from the same patient from whom the primary breast cancer cells were derived (BC160).

### Primary cell culture

Cells were cultured under standard conditions at a temperature of 37^o^C, an atmosphere enriched with 5% CO_2_ at humidity ~ 100%. Primary cells were cultivated in two different culture media, dependent on the stage of growth. Freshly disaggregated cells were cultured in 1:1 DMEM/F12 (Biowest, France), supplemented with 20% foetal bovine serum (FBS) (Biowest, France), 10 ng/mL epidermal growth factor (EGF) (Sigma-Aldrich, MO, USA), 2 mM L-glutamine (Biowest, France), 0.5 µg/mL hydrocortisone (Biowest, France), 100 U/mL insulin (Bioton S.A., Poland), 1% P/S (Merck Millipore Corporation, Germany), and 0.5% amphotericin (Biowest, France). After passages 4–5, a DMEM medium with 10% FBS and 10 ng/mL EGF was used to maintain the cell culture. The normal fibroblast cell line NF160 was cultured in DMEM medium with 10% FBS and 1% penicillin-streptomycin. All primary cell cultures were passaged using 1 mL of 0.25% trypsin-EDTA solution when confluence reached 80–90%.

### Established cell culture

T47D and MCF-7 breast cancer cell lines of the Luminal A subtype (American Type Culture Collection (ATCC), Manassas, Virginia) were used. The MCF-7 cell line was cultured in DMEM (Biowest, France) supplemented with 10% FBS, 1% P/S, and 0,01 mg/mL insulin. The T47D line was cultured in RPMI-1640 (Thermo Fisher Scientific, France) with 10% FBS and 1% P/S. Cells were passaged with 0.25% trypsin-EDTA (Merck Millipore Corporation, Germany) when they reached 80–90% confluency.

### Flow cytometry

Primary cell cultures were harvested, resuspended in PBS, and washed twice. Cells were incubated for 30 min at 4^o^C with the following antibodies: CD24 (catalogue number: B23133), CD44 (catalogue number: B37789), CD90 (catalogue number: IM1839), (Beckman Coulter Life Sciences, ID, USA), CD31 and CD45 (EXBIO, Czech Republic) diluted 1:20 in PBS. All stained cells were analysed on the Cytoflex Beckmann Coulter cytometer (Beckman Coulter Life Sciences, ID, USA). The results were analysed using FlowJo v10 (FlowJo LLC, USA).

### qPCR analysis

RNA was isolated using Direct-zol RNA MiniPrep (Zymoresearch, Irvine, CA, USA). 1 × 10^6^ cells were suspended in TRI reagent (Sigma-Aldrich, St. Louis, MO, USA) and RNA was isolated according to the manufacturer’s protocol. The iScript kit (Bio-Rad, Hercules, CA, USA) was used for reverse transcription of 1 µg of total RNA. The cDNA was amplified in a total volume of 20 µl and diluted 5 times. Next, the expression of the genes CD24, ITGB1, NANOG, POU5F1, COL1A2, SNAI1, OCLN, MMP2, TWIST1, VIM (Sigma-Aldrich, St. Louis, MO, USA) was performed using qPCR (Supplement Table 1). The human B2M gene was used as a reference gene to determine relative expression (Assay Id. 102,065). The PCR reaction was conducted in the CFX96 Touch Real-Time Detection System (Bio-Rad, Hercules, CA, USA) in 10 µl volume with PowerUp™ SYBR™ Green Master Mix (ID A25742; Life Technologies, California, USA).

**Table 1:** Comparison of evaluated methods and condition of isolated cell cultures

Property	Method 1	Method 2	Method 3	Method 4	Method 5
Days until tissue attachment	1–2	-	3–5	-	1–2
Days until colony formation	18–21	-	28	-	10–21
Number of colonies formed per well	7–10	-	2–3	-	7–12
Cell culture lifespan (number of passage)	P7-P10	-	P5	-	P7-P10+
Type and number of isolated cells per trial	CAFs (3)	-	CAFs (2)	-	BC cells (1), CAFs (2)

### Microscopy

Pictures were taken with Olympus IX83 Inverted Fluorescence Microscope (Olympus, Japan) in phase contrast with magnification of 4x or 10x.

### Statistical description

The statistical analysis was performed using Microsoft® Excel® (Microsoft Office Professional Plus 2019).

## Results

### Optimisation of primary cell line isolation methods

Based on the literature [18–21], we chose 5 protocols (Fig. 1) to find the most efficient approach for BC cell isolation. For each isolation method, we used one sample from 3 patients. Method 1 is a combination of mincing the sample and digestion in enzymes. Initially, we used collagenase IV only for 1 h, 2.5 or 24 h. We obtained a fibroblast colony after 24 h incubation, but the goal of this method was to shorten the incubation time, thus we introduced 0.25% trypsin. Cells were first immersed in the collagenase IV for 1 or 2 h and then in trypsin for 5 min or 2 min, respectively. This approach resulted in isolated fibroblasts from BC biopsy, but cells were more viable and attached to the wells quicker compared to 24 h digestion in collagenase IV only. Moreover, treatment of cells for 1 h and 5 min resulted in greater confluence than after 2 h and 2 min incubation. For this reason, we chose digestion for 1 h in collagenase IV and for 5 min in 0.25% trypsin. This method provides quick isolation since digestion lasts only an hour, and any additional mechanic force (i.e., filtration) does not disrupt the sample.

**Fig. 2:**
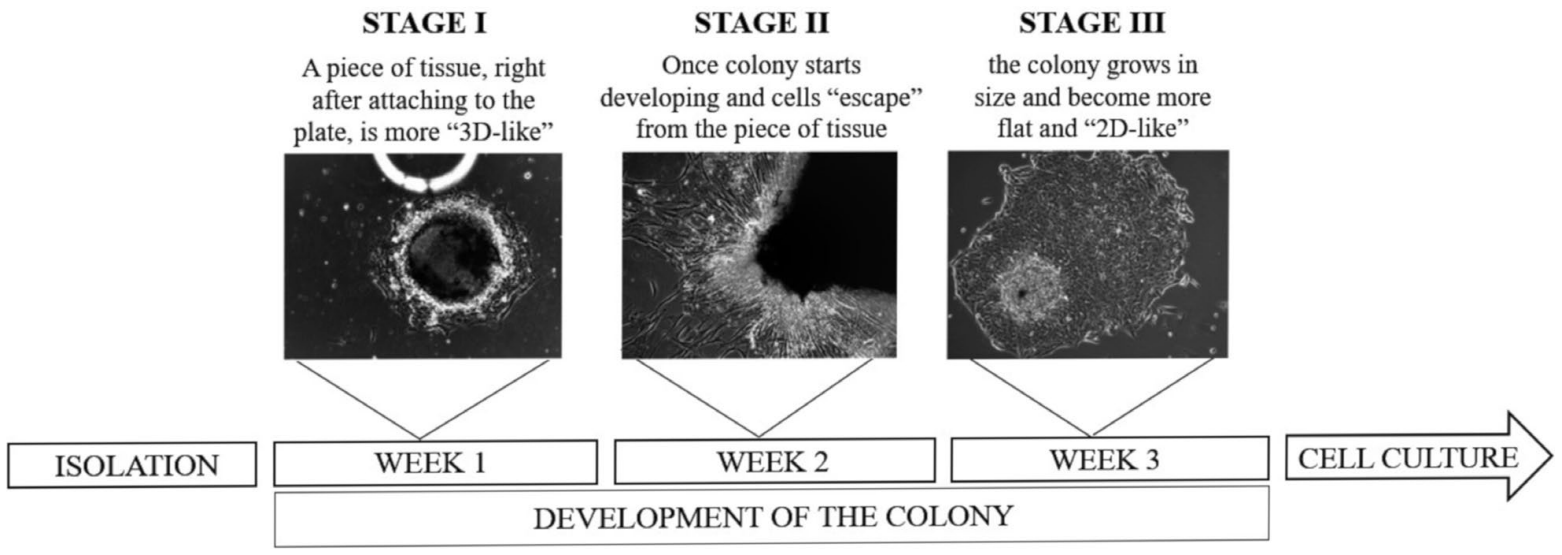
Stages of colony formation. Stage I: A piece of tumour tissue attaches to the plate; it is convex and looks more “3D-like”. Stage II: The cells’ increasing flattening and mobility are observed after attachment to the plate. Densely growing cells are visible in the colony’s centre, and spread cells at the edges. Stage III: A primary cell culture is obtained. Values are averaged based on our observations

A combination of digestion with differential centrifugation in Method 2 gave no positive results, and cells did not attach to the well in all experiments. CAFs were obtained with Method 3, however, the cells took longer to attach (3–5 days), expand (14 days) and form (28 days) (Fig. 2). The method proposed by Sigma company (Method 4) resulted in no successfully isolated primary cells and was more complicated to execute than other methods due to additional filtration and centrifugation steps. Method 5 uses a mixture of collagenase and hyaluronidase and a prolonged incubation time of up to 16 h. This approach spares the cells and enables the isolation of good-quality colonies. As a result, the primary BC cell line (BC160) was isolated. During the isolation process, colonies of the neoplastic phenotype were observed from samples isolated with Method 5, but they did not survive after several passages. Table 1 summarises the results of isolation with evaluated methods. Days until tissue attachment refers to the time until minced tissue is attached to the well. Days until colony formation indicate the time until a viable cell culture, which was able to passage, was formed.

### Primary cell culture medium optimisation

Digested tissue samples were initially cultured in a DMEM medium with 10% FBS and 1% penicillin/streptomycin. In most cases, disaggregated tissue did not attach to the plate, or the cells died quickly. Primary cells obtained through enzymatic digestion are very fragile, thus, they need growth factors and nutrients to adhere to the plate and start a colony. Changes in medium composition were introduced to increase cells’ attachment and survivability. Based on the available literature on BC isolation protocols (Table 2), a new medium for primary tissue culture was formulated (Medium 1). The medium contained 1:1 DMEM and Ham’s F-12 Nutrient Mixture medium, 20% FBS, 10 ng/mL EGF, 2 mM L-glutamine, 0.5 µg/mL hydrocortisone, 100 U/mL insulin, 1% penicillin-streptomycin, and 0.5% amphotericin B. Primary cells derived from resected tissue are prone to contamination, so besides penicillin/streptomycin solution, an additional antibiotic (amphotericin B) was introduced. The concentration of FBS was increased from 10 to 20% to favour restoring delicate cells after isolation. The mixture of hormones, such as insulin, hydrocortisone, and EGF impacts cell proliferation and cell growth. The new, well-balanced medium increased the pace of cell growth and the number of colonies after isolation and enabled the maintenance of the cells for further passages. Once the cell culture was expanded and maintained for more than 3 passages, the medium was changed to DMEM with 10% FBS and 1% penicillin/streptomycin.

**Table 2:** Different media compositions for isolation of primary cell lines from tumours

Type of medium	Serum	Antibiotics	Growth factors	Hormones	Other	References
DMEM/F12 (1:1)	10% foetal calf serum (FCS)	50 µg/mL penicillin, 0.1 mg/mL streptomycin, 2.5 µg/mL amphotericin-B, 1 µg/mL minocycline	10 ng/mL EGF	1 µg/mL insulin, 1 µg/mL hydrocortisone	10 µg/mL transferrin, 11 µg/mL ethanolamine, 50 ng/mL cholera toxin	[22]
DMEM/F12	10% FBS	1% penicillin/streptomycin	-	-	-	[23]
F12/DMEM (3:1)	FBS	-	EGF	hydrocortisone, insulin	cholera toxin, adenine, Rho-associated protein kinase (ROCK)	[24]
IMDM + epithelial cell growth supplement (EpiCGS)	10% FBS	100 U/mL penicillin, 100 µg/mL streptomycin, 250 mg/ mL amphotericin-B	EGF	-	10 µM ROCK, 2 mM L-glutamine	[25]
DMEM/F12 (1:1)	10% FCS	100 UI/ ml penicillin, 100 mg/ml streptomycin	-	2 mg/mL bovine insulin + 10 nM estradiol, 0.3 mM cortisol, 10 nM triiodothyronine, 10 ng/ml transferrin	2 mM glutamine	[26]
DMEM	10–20% FBS	-	5–15 ng/ml EGF	100 U/ml insulin	2 mM glutamine	[6]
DMEM/F12 + Geltrex®, collagen I or feeder layer	2% human serum	1% penicillin, streptomycin, 0.2% gentamycin	10 ng/ml EGF	5 µg/ml insulin, 0.32 µg/ml hydrocortisone	20 µg/ml adenine, 8.4 ng/ml CHTX, 15mM HEPES, 10 µM ROCK	[21]

### Cellular heterogeneity determines successful isolation

**Fig. 3:**
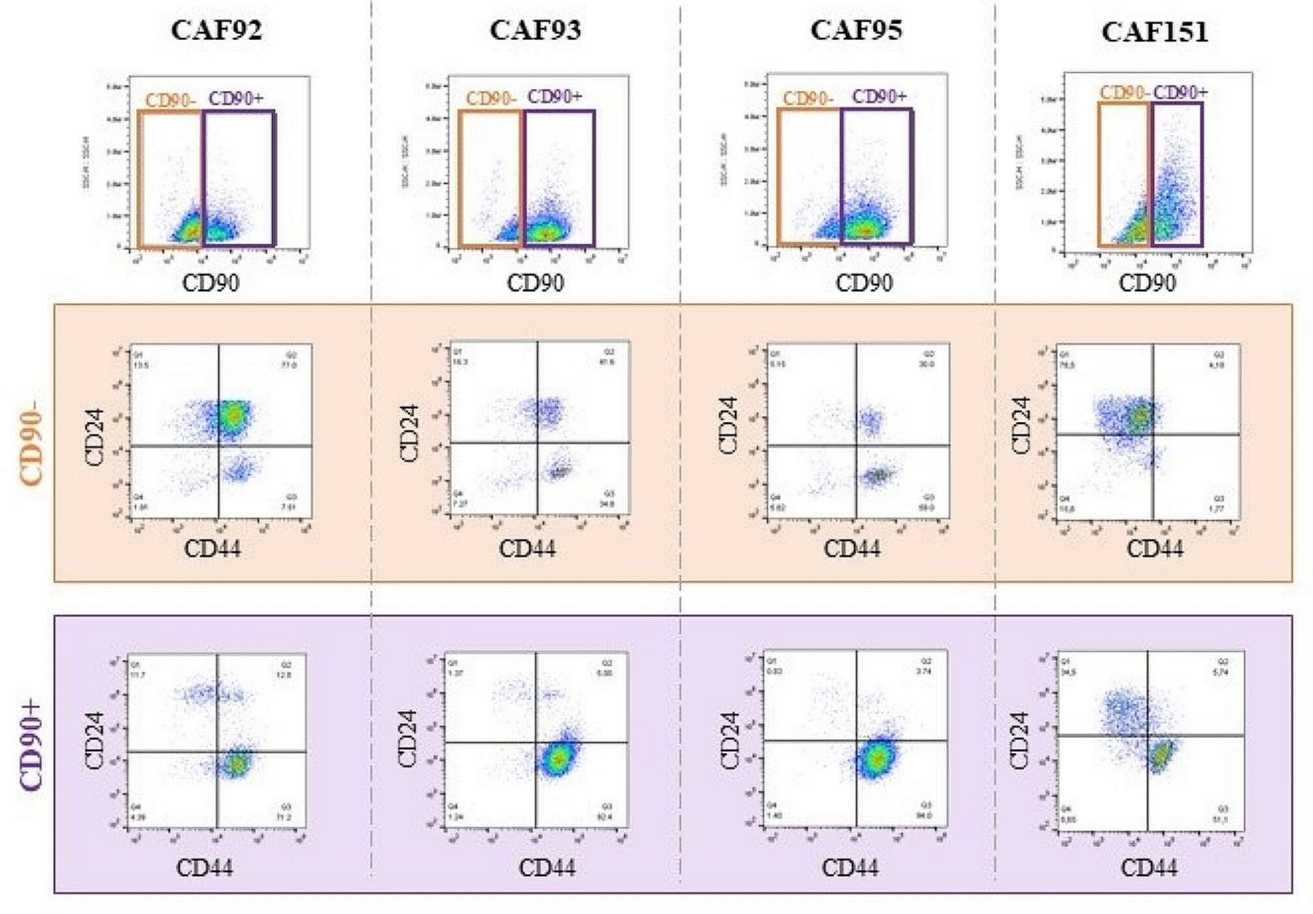
Cellular heterogeneity of primary cell populations according to basic phenotyping. Cells were stained with CD90, a marker characteristic of fibroblast origin, and populations were evaluated by flow cytometry. In each sample, CD90 + and CD90-cell heterogeneity was observed. In the second and third rows, the CD90^+/−^ cells were characterised using CD24 and CD44 to assess their stem-like phenotype

The basic phenotyping was performed when primary cells stably proliferated. CD90 was used as a marker to assess the fibroblast origin of cells. Moreover, cells were stained with CD24 and CD44 to assess the cancer stem-like phenotype. The heterogeneity was indicated by the presence of CD90-positive (fibroblast-like) and CD90-negative (cancer cell-like) populations. Depending on the proportion of CD90+/- cells, the CD24 and CD44 expression levels highlighted the variations between different patient samples (Fig. 3).

**Fig. 4:**
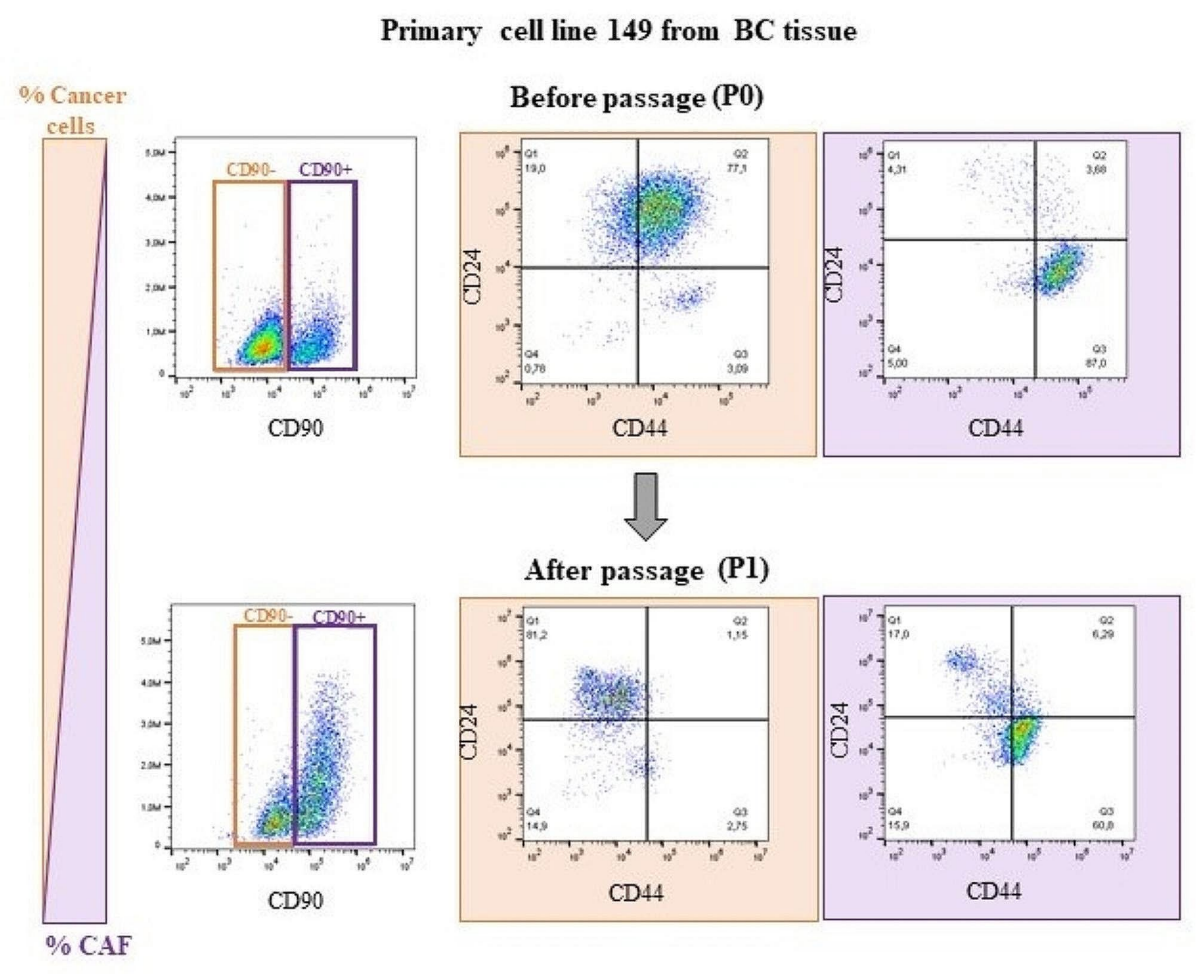
The changing phenotype of cell lines after passaging. Due to the heterogenic composition of cells in the BC tissue, the percentage of different cell populations varies. During the isolation process, the passage caused the reduction of the population of neoplastic cells, resulting in the derivation of the CAF cell line. Cells were stained with CD90, CD24 and CD44 and assessed with flow cytometry

The primary cells’ ability to re-adhere and divide significantly decreased after passage, while fibroblasts [27] continued to grow and divide during passaging and repelled BC cells (Fig. 4).

**Fig. 5:**
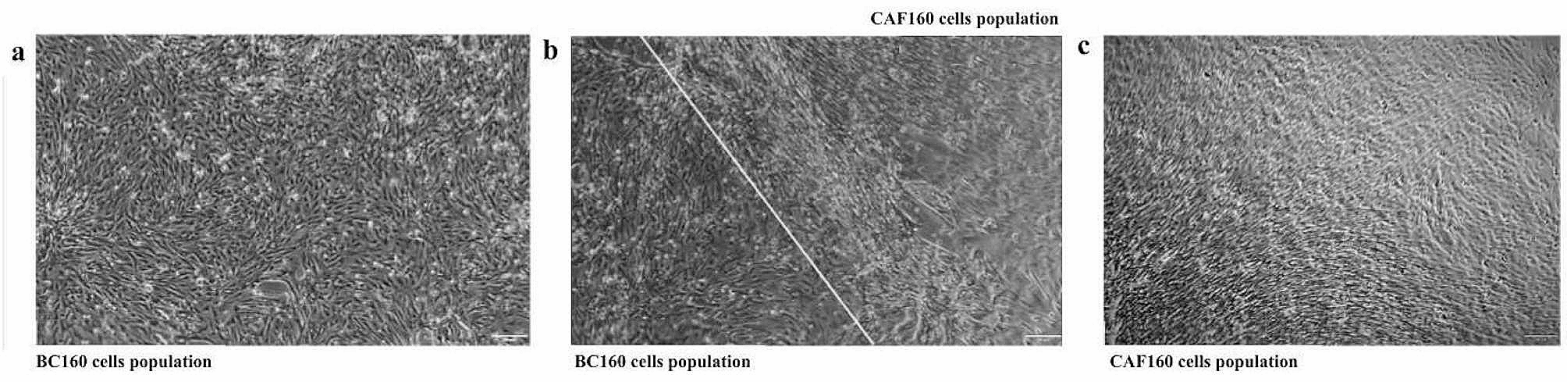
Populations of cells isolated from a biopsy sample of patient no 160. (**a**) Cells from primary cell culture BC160, homogenous population. (**b**) Two cell populations are present after plating cells from one biopsy; the white line demarks a boundary between distinct populations – on the left cancer cells population, on the right CAF population. (**c**) Cells from primary cell culture CAF160, homogenous population. Scale: 200 μm, magnification 4x

A primary cell culture heterogeneity was very frequently observed. Populations of BC cells and CAFs were seen in the same well after isolation from a biopsy sample of patient number 160 (Method 5) (Fig. 5). The type of cell line established from a tissue depended on the proportion of BC and CAF cells in the well. When the number of CAF cells was higher than BC cells, CAFs overgrew BC cells with each passage. Hence, the CAF primary cell culture could be established from the same patient.

### Obtaining a primary BC cell line

From 15 samples processed using Method 5, we established one stable, primary BC cell line from a 75-year-old patient. Primary BC cell line (BC160) of Luminal A subtype was obtained from the right breast, tumour grade NHG2, and isolated as described in the methods section. Histopathologically, the expression of oestrogen and progesterone receptors was positive, while human epidermal growth factor receptor 2 (HER2) and E-cadherin were negative. Expression of Ki67 was observed in 5% of cancer cells in examined biopsy. Primary cell line NF160 was obtained from a skin tissue sample of the same patient (same breast) and served as a non-neoplastic control.

#### Phenotyping of isolated primary BC cell line

**Fig. 6:**
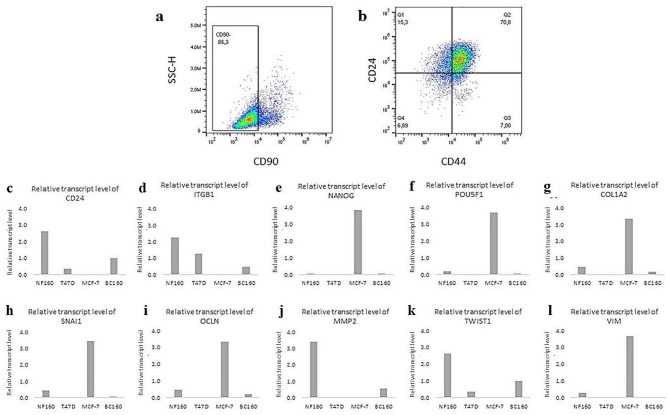
(A, B) Phenotyping of BC160 cells. The BC cell line isolated from the patients was characterised using CD90, CD24, and CD44 to verify its cancer phenotype. A: Distribution of CD90^−^ population, B: Distribution of CD24 and CD44 positive/negative cells. (C-L) Relative transcript levels of CD24 (**A**), ITGB1 (**B**), NANOG (**C**) and POU5F1 (**D**), COL1A2 (**E**), SNAI1 (**F**), OCLN (**G**), MMP2 (**H**), TWIST1 (**I**), VIM (**J**) in BC160, NF160, T47D, and MCF-7 cell lines

Flow cytometry was performed to confirm the origin of the BC160 cell line. An appropriate gating strategy was applied to exclude debris, dead cells, and doublets. Three markers were analysed: CD90 for fibroblastic origin [28], CD24 for the epithelial origin [29], and CD44 to examine tumourigenicity [30]. Flow cytometry revealed that BC160 was predominantly negative for CD90 (Fig. 6a), and to a large extent, it exhibited the CD24^+^/CD44^+^ subtype (Fig. 6b).

The qPCR reaction was performed to determine the expression of genes specific to BC in the BC160 cell line. The BC160 cell line was compared to other Luminal A cell lines, T47D and MCF-7, and to the fibroblast NF160 primary cell line obtained from tissue from the same patient. Examination of the BC160 revealed a lack of VIM expression and very weak expression of NANOG, POU5F1, COL1A2, and SNAI1, which are highly expressed in MCF-7 cells (Table 3). However, BC160 showed a moderate expression of the CD24 and TWIST1 genes not expressed in MCF-7. The T47D cell line has expression levels of examined genes similar to BC160 (Fig. 6c-l). Only BC160 and NF160 showed MMP2 gene expression, which might be characteristic for this patient.

**Table 3:** Relative gene expression of BC160, NF160, T47D, and MCF-7 cell lines

Cell line/Gene	CD24	ITGB1	NANOG	POU5F1	COL1A2	SNAI1	OCLN	MMP2	TWIST	VIM
NF160	+++	+++	-/+	+	+	+	+	+++	+++	+
BC160	++	+	-/+	-/+	+	-/+	+	+	++	-
T47D	+	++	-	-	-	-	-	-	+	-
MCF-7	-	-	+++	+++	+++	+++	+++	-	-	+++

Relative expression: - lack of expression, -/+ very weak, + weak (< 1), ++ moderate (1–2), +++ high (> 2)

## Discussion

One of the biggest concerns in BC research is obtaining clinically relevant results from in vitro studies of treatment methods and responses to therapies, which are often conducted on primary cell lines. Primary cell lines obtained from patient tissues are a helpful tool for studying hormone responsiveness and the effect of treatment on tumour cells. Thus, short-term cultivation of primary cells from tumours has great potential for developing personalised cancer therapy since the isolated cells preserve characteristic neoplasm features [14]. To obtain the primary cell culture model, the crucial step is the appropriate treatment of the tissue and isolation of the cells. Many protocols and approaches differ in conditions, reagents, and handling. A preferable method is the enzymatic digestion of biological material. Different enzymes should be used depending on tissue structure, including collagenase, hyaluronidase, dispase, or DNase [16]. Cell isolation with collagenase and hyaluronidase is a popular approach for BC tissue [6, 12, 13]. The effect and efficiency of digestion change depending on the enzyme concentrations and the incubation time. Zubeldia-Plazaola et al. assessed the efficacy of different concentrations of collagenase and hyaluronidase. The cancer tissue was digested overnight at 1.6 and 0.14 mg/mL or 4–6 h, with both collagenase and hyaluronidase concentrations of 2 mg/mL. This study confirmed the superiority of slow digestion due to the higher number of viable cells and the less harmful conditions compared to fast digestion [13]. Another approach utilising digestion with hyaluronidase and collagenase was proposed by Janik and colleagues, who minced and dissolved a breast tumour tissue in hyaluronidase/collagenase solution for 16 h, at 37 ºC on the shaker. This step was followed by gently pipetting the tissue slurry with 0.25% trypsin and then with a mixture of dispase (5 units/mL) and DNase I (0.05 mg/mL), and cells were seeded on a feeding layer or Geltrex. The authors obtained 72% effectiveness [21].

In our study, five protocols were tested and optimised to isolate primary BC cell lines. Method 1 and Method 5 were the most favourable for cells, producing the most colonies in a short time and consisting of viable cells. Both methods did not use additional mechanical approaches such as vigorous pipetting, multiple centrifugations, or filtering, which prevented cells from additional disturbances. Only Method 5 gave rise to a stable primary cancer cell culture. Method 5 uses a mixture of collagenase and hyaluronidase, which is more favourable considering breast tissue composition. Moreover, Method 5 involves prolonged incubation and does not harm the cells since the enzyme mixture is diluted in a culture medium. The other tested protocols (Methods 2, 3, 4) use combinations of enzymatic digestion with various mechanical disruption methods. Method 2, utilising differential centrifugation, was supposed to enable the derivation of epithelial and fibroblast fractions, but we did not obtain any cell populations. Method 3 has led to the isolation of two CAF cell lines; however, it took them longer to attach to the surface and expand. Similarly, Method 4 gave no successful results and was complicated to execute. These results suggest that mechanical disruption is too aggressive and harmful for primary cells, making it challenging to obtain viable colonies.

An important factor influencing cell viability is the isolation of the biological material. It might be gathered via resection, fine-needle aspirates or core biopsy [14]. Most of the biological is derived during surgeries (resection, mastectomy) instead of biopsy [6, 8–13]. Considering it, obtaining primary cell lines might be more readily possible if the material available for isolation is derived by a method other than biopsy. Nevertheless, the isolation of cell lines from biopsy could be advantageous for evaluating cells before choosing a treatment. This approach would enable selecting the best therapy for a given patient and applying a more personalised approach, as patient-derived explants (PDE) are often used for this purpose [31].

Depending on the aim of the study, the presence of other TME cells can be considered an advantage or disadvantage. TME cells provide a unique environment, but also [7] it is complicated to obtain a pure cancer cell line. In this study, we often observed the high heterogeneity of isolated populations, which resulted in the overgrowth by faster-dividing CAFs and inhibition of BC cell growth (Figs. 3 and 4). The viable cells of the neoplastic phenotype were frequently observed, however, they died after passaging. The great importance of successful isolation lies in the biopsy itself. The localisation within the tumour from which the biopsy was derived might impact the further composition of the primary culture. Tumours spatially vary in cellular composition, which can result in a higher content of some groups of cells. Immunohistochemistry staining and spatial transcriptomics show that cells within tumour grow in clusters rather than a uniform mixture of cells [32, 33]. CAFs often reside in the so-called invasive tumour front [34, 35], and cancer cells prefer the close vicinity of the hypoxic core due to metabolic benefits [36].

Method 5 was a convenient and effective approach to isolating BC primary cells. Using this method, we were able to isolate one stable primary cancer cell line, which we subsequently characterised. Gene expression was assessed by RT-qPCR, which revealed characteristic features of the BC160 primary cell culture isolated from a patient with the BC of Luminal A subtype. BC160 shows a gene expression pattern similar to the T47D cell line compared to other established Luminal A cell lines. The expression of CD24 may suggest the stem-like phenotype BC160 and T47D, which was also observed in NF from the same patient. CD24 can also indicate a mammary epithelial origin of BC160 and T47D cells [29] since T47D originates from infiltrating ductal carcinoma and is an epithelial cell line. Another characteristic of BC160 is the expression of extracellular matrix (ECM) remodelling genes, such as ITGB1 and MMP2 [37, 38]. Integrin signalling dysregulation alters cell-cell and cell-ECM interactions and facilitates breast cancer growth by inducing chemoresistance and metastasis [39]. Moreover, ITGB1 is upregulated during epithelial-mesenchymal transition (EMT), which correlates with BC’s progression [40]. The cell line BC160 expresses matrix metalloproteinase 2 (MMP2), an enzyme-degrading ECM element that leads to uncontrolled cell proliferation and invasion, cell death inhibition, and cell differentiation loss [41]. MMP2 is investigated as a BC biomarker indicating cancer prognosis as its expression correlates with lymph node metastasis [42]. Another gene expressed in examined cell line BC160 is Twist1, a crucial player during EMT, which also correlates with BC invasion [43]. Additionally, a study by Wafai et al. suggests a concurrent expression of Twist1 and upregulation of ITBG1/2 in Luminal A tumours [40]. BC160 cells show weak occludin (OCLN) expression, a migration marker. It is a membrane protein found in tight junctions (TJs). Pieces of evidence suggest that the TJs are essential structures that cancer cells must overcome to migrate. Low or complete lack of OCLN expression leads to loss of cell structure junctions that facilitate BC progression and metastasis [44]. Overall, the results suggest a substantial contribution of genes related to ECM remodelling and intracellular junctions.

The biological material derived from biopsy consists of the tumour, tissues, and TME cells, significantly impacting the acquisition of pure, homogenous cell culture. The other obstacle is choosing a method appropriate for isolation, followed by maintaining a culture consisting only of the cells of interest. The challenge for future experiments is the elaboration of methods for the separation of heterogeneous cultures. We have made efforts to divide cancer cultures from TME cells by single-cell assay and differential digestion times during passage, however, it proved inefficacious.

It is essential to publish more reports concerning primary cell lines that would allow the results and isolation methods to be compared, as there is no systematic breakdown or categorisation of methods. Research groups work on biological material derived from different methods, which might influence isolation success. The source of biological material and its impact on primary culture requires further exploration, and it is challenging to observe reproducibility between published results since the methods always differ in the kind of enzyme used, incubation time, and further isolation steps, which hampers a search for a suitable, standardised isolation method. Formulating isolation methods and establishing the BC primary cell line is still a significant challenge, and its achievement depends on numerous factors.

## Conclusions

The increasing popularity of primary cancer cells among oncology researchers across the globe requires a reliable method for their isolation and handling. The availability of primary cell lines obtained from a specific patient may improve research related to personalised therapy. The approach described in this study allowed for the effective isolation of a viable primary BC cell line, which maintained its molecular features throughout the culture. The enzymatic composition of the solution used to isolate primary cell lines and the time of digestion and concentration is crucial for successful isolation. In the age of molecular diagnostics, testing pharmaceuticals or drug variations directly on patient tissue samples appears to be a powerful method of improving anti-cancer therapies.

### Electronic supplementary material

Below is the link to the electronic supplementary material.

Supplementary Material 1

## Data Availability

The data supporting this study’s findings are available from the corresponding author, upon reasonable request.

## Abbreviations

BC: breast cancer
TME: tumour microenvironment
CAFs: cancer–associated fibroblasts
NFs: normal fibroblasts
GCDFP15: gross cystic disease fluid protein–15
ER: oestrogen receptor
PR: progesterone receptor
HER2: human epithelial growth factor 2
BSA: bovine serum albumin
EGF: epidermal growth factor
FBS: foetal bovine serum
OCLN: occluding
