# Supplementary material for: Navigating challenges: optimising methods for primary cell culture isolation
Source: Cancer Cell Int. 2024 Jan 11;24:28. doi: 10.1186/s12935-023-03190-4 (PMC10785493; doi:10.1186/s12935-023-03190-4)
Supplement: Supplementary file 1 — Supplementary Material 1 [file 12935_2023_3190_MOESM1_ESM.docx]

**Supplement Table 1.** List of primers used for real-time PCR analysis

| Gene | Primer | Sequence (5'-3') |
| --- | --- | --- |
| CD24 | Forward | ATGGGCAGAGCAATGGTTG |
|  | Reverse | CCAGTTGTTGTTTCACTGGAAT |
| ITGB1 | Forward | CAAGTTGCAGTTTGTGGATCA |
|  | Reverse | AACAATGCCACCAAGTTTCC |
| NANOG | Forward | ATGCCTCACACGGAGACTGT |
|  | Reverse | AAGTGGGTTGTTTGCCTTTG |
| POU5F1 | Forward | CTTCGGATTTCGCCTTCTC |
|  | Reverse | CTTAGCCAGGTCCGAGGAT |
| COL1A2 | Forward | TGATGGAAAAGGAGTTGGACTT |
|  | Reverse | CAGGTCCTTGGAAACCTTGA |
| SNAIL1 | Forward | GCTGCAGGACTCTAATCCAGA |
|  | Reverse | ATCTCCGGAGGTGGGATG |
| OCLN | Forward | CACTATGAGACAGACTACACAACTGG |
|  | Reverse | TTGATCTGAAGTGATAGGTGGATATT |
| MMP2 | Forward | ATGCCGCCTTTAACTGGAG |
|  | Reverse | GGAAGCCAGGATCCATTTTC |
| TWIST1 | Forward | GGGCCGGAGACCTAGATG |
|  | Reverse | TTTCCAAGAAAATCTTTGGCATA |
| VIM | Forward | CCGCTAGGAGCCCTCAAT |
|  | Reverse | ACTGGCTCCCGGAGAAGA |
